# MiR-20a Promotes Cervical Cancer Proliferation and Metastasis In Vitro and In Vivo

**DOI:** 10.1371/journal.pone.0120905

**Published:** 2015-03-24

**Authors:** Shan Zhao, Desheng Yao, Junying Chen, Nan Ding, Fei Ren

**Affiliations:** 1 Department of Gynecologic Oncology, the Affiliated Tumor Hospital of Guangxi Medical University, Nanning, China; 2 Department of Gynecology, Qianfoshan Hospital Affiliated to Shandong University, Jinan, China; The University of Hong Kong, CHINA

## Abstract

MicroRNAs (miRNAs) are small, non-coding RNAs that are critical regulators of various diseases. MicroRNA-20a (miR-20a) has previously significantly altered in a range of cancers. In this study, we detected the relationship between miR-20a and the development of cervical cancer by qRT-PCR, we found that the expression level of miR-20a was significantly higher in cervical cancer patients than in normal controls, the aberrant expression of miR-20a was correlated with lymph node metastasis, histological grade and tumor diameter. Then we successfully established the stable anti-miR-20a cervical cancer cell lines by lentivirus. Inhibited miR-20a prevented tumor progression by modulating cell cycle, apoptosis, and metastasis in vitro and in vivo. TIMP2 and ATG7 were proved to be direct targets of miR-20a, using luciferase assay and western blot. These results indicate that miR-20a suppresses the proliferation, migration and invasion of cervical cancer cell through targeting ATG7 and TIMP2. Our results support the involvement of miR-20a in cervical tumorigenesis, especially lymph node metastasis. We propose that miRNAs might be used as therapeutic agent for cervical cancer.

## Introduction

Cervical cancer is the second most common type of cancer in women all over the world, which is a leading cause of cancer death, resulting in about 300,000 deaths each year[1]. Most cervical cancer patients receive standard radiotherapy and chemotherapy. However, clinical outcomes vary significantly. According to the world, there were about 500,000 new cases each year, 85% of the pathologic types were squamous cell carcinoma. Although the cervical cytology screening benefits a lot in early detection and early treatment in recent decades, cervical cancer morbidity rates remain high, and there were more and more young cases recently. So many researchers devote themselves to find pathogenesis and more effective tumor therapy. microRNAs (miRNAs) are small non-coding RNAs of approximately 21–25 nucleotides (nt) and act as post-transcriptional regulators of gene expression [2]. These small molecules have been found to regulate genes involved in diverse biological processes such as cell proliferation, development, differentiation, apoptosis and others. Numerous of studies have shown that alterations in miRNAs synthesis in human cancers are often related to tumor development, progression and metastasis[3]. Our early experiments by hybridization arrays compared the microRNA expression profile between normal cervical tissues and cervical squamous cell carcinomas tissues, 89 miRNAs were examined, and miR-20a was up-regulated significantly. MiR-20a was known to belong to the miR-17-92 cluster, which is the most extensively studied cluster that has an oncogenic function [4]. In this study we focus on miR-20a which could be a promising starting point for developing future miRNA-based cervical cancer therapy.

## Materials and Methods

### Ethical Statement

The consent procedure and the conductance of this study were approved by the Ethics Committee of the Guangxi Medical University. The study was carried out in compliance with the principles laid down in the Helsinki Declaration. Data were collected anonymously. Verbal informed consent was obtained from all subjects, witnessed, and formally recorded for every survey. Written consent for using the samples for research purposes was obtained from all patients prior to surgery. Animal experiment was carried out in strict accordance with the Guide for the Care and Use of Laboratory Animals of the U.S. National Institutes of Health, and the study protocol was approved by the Committee on the Ethics of Animal Experiments of Guangxi Medical University.

### Patients and Samples

Cervical tissue samples were collected from the department of gynecologic oncology, the Affiliated Tumor Hospital of Guangxi Medical University between 2010 and 2011. Eighty cervical cancer samples of international Federation of Gynecology and Obstetrics (FIGO) stageⅠ-ⅡA were obtained from patients who underwent surgical treatment. Twenty samples of stageⅡB-Ⅳ were got from cervical biopsy. All samples were squamous cell carcinoma. No previous local or systemic treatment had been conducted on these patients before the operation or biopsy. LNM was confirmed in patients of stage Ⅰ-ⅡA which we used operation for the first treatment. The median age for patients was 49 years with a range from 25 to 69 years. Normal cervical epithelium samples were collected from 120 patients who had hysterectomy for benign disease. The mean age for control subjects was 45 years, ranging from 33 to 57 years. The tissues were frozen in liquid nitrogen immediately after surgical removal and stored at −80°C until use.

### Quantitative Real-Time Polymerase Chain Reaction

RNA was extracted from frozen fresh cervical cancer tissues, normal cervical epithelium tissues and cervical cancer cells using the miRcut miRNA isolation kit(Tiangen, China) according to the manufacturer’s instructions. The reverse-transcription reactions were carried out using an MiraMasTM Kit (Bioo scientific, USA), which contains poly (A) polymerase used for polyadenylation of miRNA. qRT-PCR was performed using a standard SYBR Green PCR kit (Takara, Japan).The primers were synthesized (Shanghai GenePharma, China) as follows: miR-20a forwards primer: TAC GAT AAA GTG CTT ATA GTG CAG GTA G. U6 forwards primer: ATT GGA ACG ATA CAG AGA AGA TT. Universal reverse primer: GTC CTT GGT GCC CGA GTG. The 20 μl PCR mixture consisted of 12.5 μl SYBR Green supermix, 3.5 μl RNase-free water, 1 μl forward primers, 1 μl reverse primers, and 2 μl reverse transcribed product. The reaction conditions were 40 amplification cycles of 95°C for 3 min, 95°C for 12 s, and 62°C for 50 s using a BIO-chromo4 (Bio-Rad, USA) quantitative Real-Time PCR System. U6 was used as references for miRNAs. Each sample was analyzed in triplicate. Comparative threshold cycle (CT) method-fold change (2-ΔΔCT) was used to analyze relative changes.

### Cell Culture

The CC cell lines SiHa, HCC94, C33A and Caski were obtained from central laboratory of the Affiliated Tumor Hospital of Guangxi Medical University. These cells were maintained at 37°C in an atmosphere of 5% CO2 in RPMI-1640, with 10% fetal bovine serum.

### Establish the stable anti-miR-20a cervical cancer cell lines by lentivirus

The lentiviral vectors with miRNAs that were based on the miR-20a framework was constructed by the GeneChem Company (Shanghai, China). Briefly, the double-stranded oligonucleotide encoding anti-miRNA and negative control were annealed and inserted into the linearized eukaryotic pFU-GW-009 vector (GeneChem). All these vectors were confirmed by sequencing. The recombinational vectors and the packaging vectors (pHelper 1.0 and pHelper 2.0) (GeneChem) were co-transfected into 293T cells (Invitrogen, Carlsbad, CA, USA) with Lipofectamine2000 (Invitrogen). The culture supernatants were collected at 48 hours after transfection, concentrated. All the lentiviral vectors expressed enhanced green fluorescent protein (GFP), which allowed for tittering and measuring their infection efficiency in infected cells. The cells were divided into three groups according our objectives: a: without infection of virus (NC); b: infection of control virus (NC-LV); c: infection of anti-miR-20a virus (anti-miR-20a-LV).

### Cell biology function in vitro

#### Cellular viability assay

The capacity for cellular proliferation was measured with the 3-(4,5-dimethylthiazol-2-yl)-2,5-diphenyltetrazolium bromide (MTT) assay. Twenty-four hours after transfection, 1×10^4^ cells were seeded into 96-well microtiter plate for 24, 48, 72, and 96 h, respectively. Then, the cells were incubated with 20 μl of MTT (5 mg/ml, PH = 7.4) for 4 h at 37°C and 150 μl of dimethyl sulfide was added to solubilize the crystals for 20 min at room temperature. Optical density (OD) was measured at a wavelength of 490 nm. All experiments were performed three times and were calculated using average results, which we used to draw the growth curves. Growth inhibition rate was calculated as following: (AC−AT)/AC×100% (AC = absorbance value of the NC and AT = absorbance value of the experimental group).

Cell cycle assay. Cells were fixed in 70% ethanol for 2 h at 4°C. After washing with PBS, cells were treated with RnaseA (50 μg/ml) and stained with propidium iodide (25 μg/ml) for 30 min at 37°C. Samples were analyzed using an flow cytometer and distribution of cell-cycle phases was determined using Modfit Software (BD Biosciences). The proliferative index was calculated as the percentage of cells in S/G2/M-phase.

#### Cell apoptosis assay

Cells were harvested and stained with annexin V-PE and 7AAD using the ANNEXIN V-PE Kit (Beckman) according to the manufacturer’s protocol and subjected to flow cytometric analysis.

#### Invasive and migration assay

50,000 cells were seeded into transwell insert (Corning) supplemented with 1640 with 10% serum. The bottom side of transwells was filled with 1640 with 20% serum. For transmigration assay, Matrige l(BD Biosciences) was seeded into transwell insert and allowed to grow to confluence for 1 day. 50,000 cells were seeded into transwell inserts supplemented with 1640 with 10% serum. The bottom side of transwells was filled with 1640 with 20% serum. After 24 hours, membranes were stained using hematoxylin-eosin staining, cells were counted under the fluorescent microscope. This experiment was performed independently three times in duplicates.

#### Colony formation assay

Approximately 500 cells were placed in a fresh 6-well plate for another 12 h and maintained in RMPI 1640 containing 10% FBS for 2 weeks. Colonies were fixed with methanol and stained with 0.1% crystal violet in 20% methanol for 15 min. Cells were counted under the fluorescent microscope. This experiment was performed independently three times in duplicates.

### In vivo assays for tumor proliferation

Twenty four 6-week-old weighing 19–21 g immunodeficient beige/nude/xid nu/nu female mice were purchased from Guangxi Medical University, and maintained under pathogen-free conditions with irradiated chow. In our experiment, 2 ×10^6^ cells of different groups in 0.2 mL PBS was subcutaneously injected into the trunk of 24 mice, leading to the formation of a tumor per animal. 0.2ml chloral hydrate was intraperitoneal injected to take fluorescent images after anesthesia when the diameter of tumor was 2 cm. Draw the growth curve. Tumor growth was monitored by the tumor volume, which was calculated as described: Volume (mm^3^) = width^2^ (mm^2^) × length (mm) /2.

### Western blot

Cellular proteins were prepared using cell lysis buffer (50 mM Tris-HCl, pH 8.0, 1% NP-40, 2 mM EDTA, 10 mM NaCl, 2 mg/ml aprotinin, 5 mg/ml leupeptin, 2 mg/ml pepstatin, 1 mM DTT, 0.1% SDS and 1 mM phenylmethylsulfonyl fluoride). Equal amounts of protein (50 μg) were separated by 10% SDS PAGE and then transferred to nitrocellulose membranes (NY, USA) by electroblotting. The membranes were blocked with 5% BSA in TBST (10 mM Tris-HCl, pH 8.0, 150 mM NaCl, and 0.05% Tween 20) for 1 h, and then incubated with primary antibodies (Santa Cruz) overnight at 4°C before subsequent incubation with second antibody (BD) for 1 h at 37°C. Protein was visualized using enhanced chemiluminescence reagent (Santa Cruz).

### Luciferase assay

The miR-20a binding sites from 3′ UTR ATG7/TIMP2 or mutant 3′ UTR were cloned into the pGL3 reporter luciferase vector (GeneChem). For reporter assay, 100 nM miR-20a mimic or control miRNA was co-transfected with 0.1μg of the pGL3–3’UTR wildtype or mutant plasmid DNAs into SiHa cells in 96-well plates using Lipofectamine 2000. Luciferase activity was measured 48 hours posttransfection as described previously.

### Statistical analysis

All data were processed using PASW Statistics 16. Data were presented as mean ± SEM. using t tests for 2-group comparisons. While the results did not display normal distribution, we chose to analyze the data with non-parametric methods. (Mann-Whitney U test between two groups and Kruskal-Wallis H test for three or more groups). A *P* value less than 0.05 is considered statistically significant.

## Results

### MiR-20a is up-regulated in cervical Cancer

**Fig 1:**
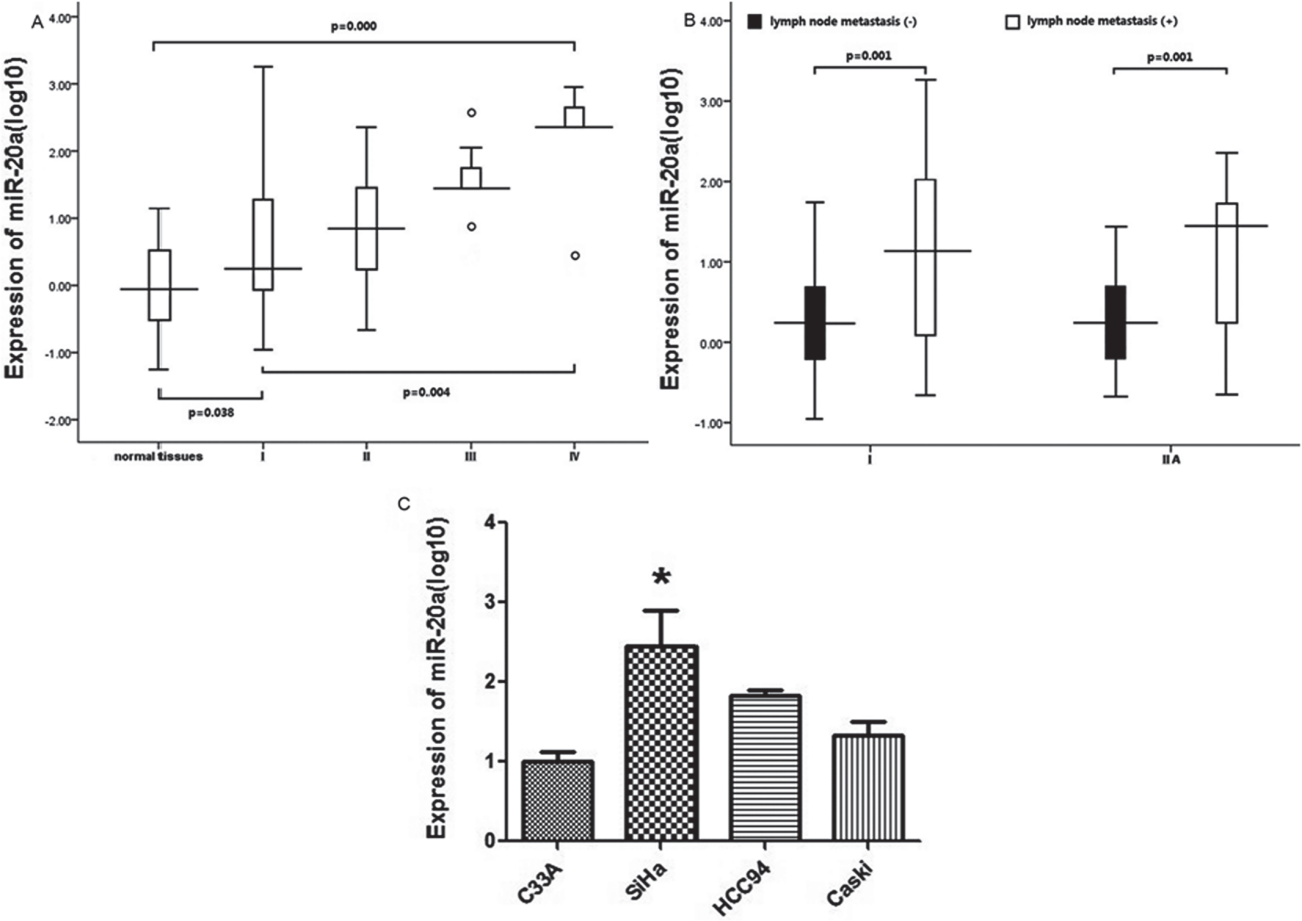
Expression of miR-20a in 100 patients with cervical cancer. A: MiR-20a was measured by qRT-PCR. Datas were presented as log10 of fold-change. The Mann–Whitney test was performed to examine the difference between normal controls and cervical cancer patients, Kruskal–Wallis H test was used to define the difference among stages I–IV of cervical cancer patients. B: We compared the expression between patients which had lymp node metastasis or not in the same stage. C: The expression levels of miR-20a was detected by qRT–PCR in cervical cancer cell lines (SiHa, HCC94, C33A and Caski). *P*-value<0.05 was considered significant.

Using a qRT-PCR method, miR-20a levels were detected in 100 cervical cancer tissues and 120 normal cervical tissues, as well as cervical cell lines. Among the 100 patients with cervical cancer, the data reported in Fig. 1A shows miR-20a is definitely up-regulated in the impaired tissue with a median of 6.92, suggesting that increase of miR-20a was a frequent event in cervical cancer. Patients with up-regulated expression of miR-20a tended to have LNM (*P* = 0.001) as in Fig. 1B, increased tumor sizes (*P* = 0.013, Mann–Whitney U test), advanced stage (*P* = 0.004, Kruskal-Wallis H test), and advanced histological grade (*P* = 0.027, Mann–Whitney U test) of cervical cancer. These results suggest that the miR-20a might play a critical role in the cervical cancer metastasis and progression.

Moreover, the expression levels of miR-20a were detected by qRT–PCR in cervical cancer cell lines (SiHa, HCC94, C33A and Caski) (Fig. 1C) which was highest in SiHa cell line.

### MiR-20a facilitates Cervical cancer Growth and Metastasis in vitro

**Fig 2:**
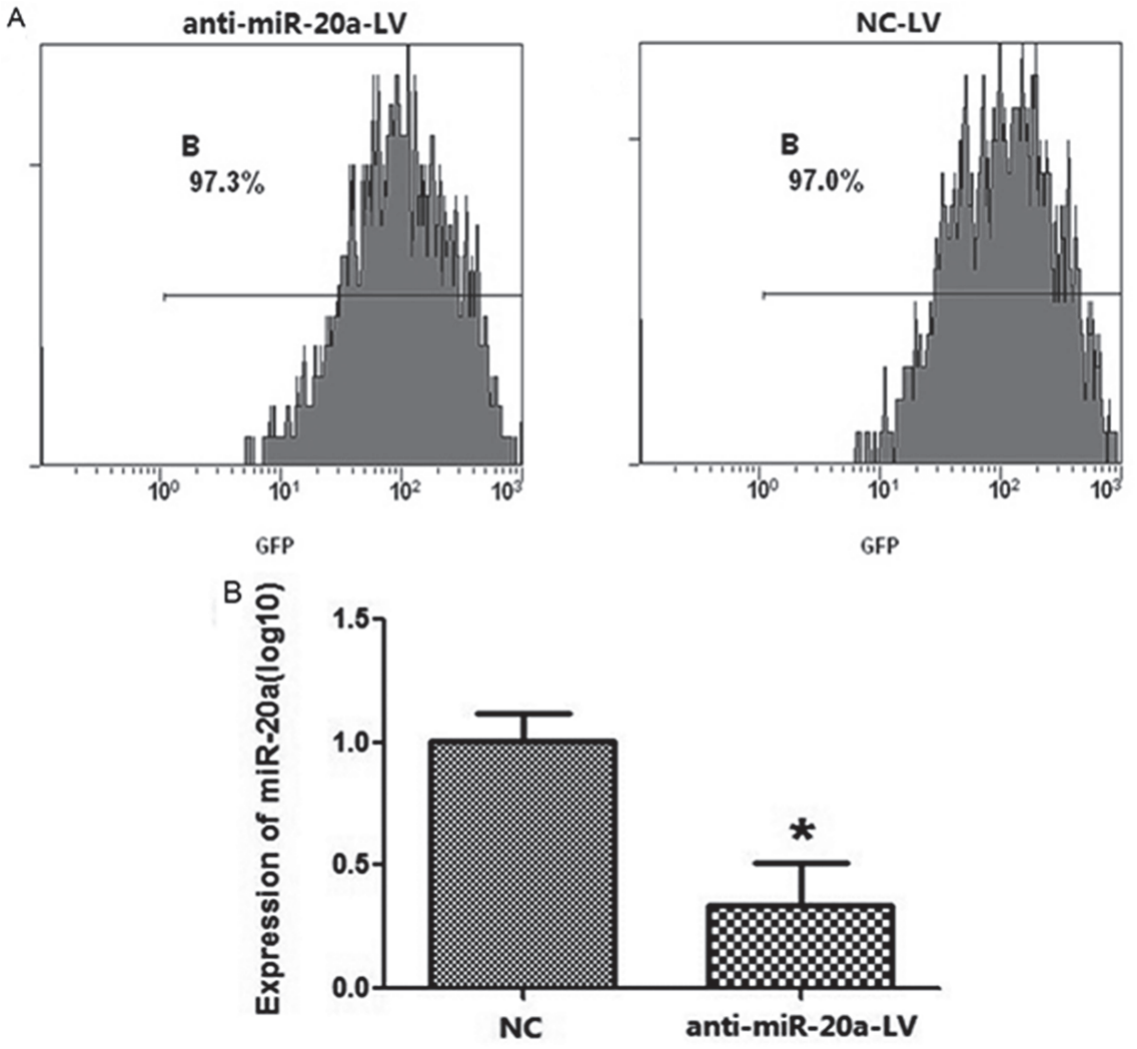
Establish the stable anti-miR-20a cervical cancer cell lines by lentivirus. A: GFP labeling flow cytometry analysis indicated that the percent of GFP positive cells were about 97% in stable lentivirus-infected SiHa cell line. B: MiR-20a was lower expressed in stable anti-miR-20a SiHa cell line while the expression level of miR-20a was still very high in cells infected with negative control.

Noting the correlation between up-regulated miR-20a levels and cervical cancer progression, we investigated the effect of miR-20a on the growth, migration and invasion abilities of cervical cancer cell lines. SiHa cell line was infected with either anti-miR-20a or control lentivirus and establish the stable anti-miR-20a SiHa cell line (Fig. 2A). Compared with the expression of control group, miR-20a was significantly down-regulated in the SiHa cell line after infection with anti-miR-20a-LV (Fig. 2B). The cells were divided into three groups according our objectives: a: without infection of virus (NC); b: infection of control virus (NC-LV); c: infection of anti-miR-20a virus (anti-miR-20a-LV).

**Fig 3:**
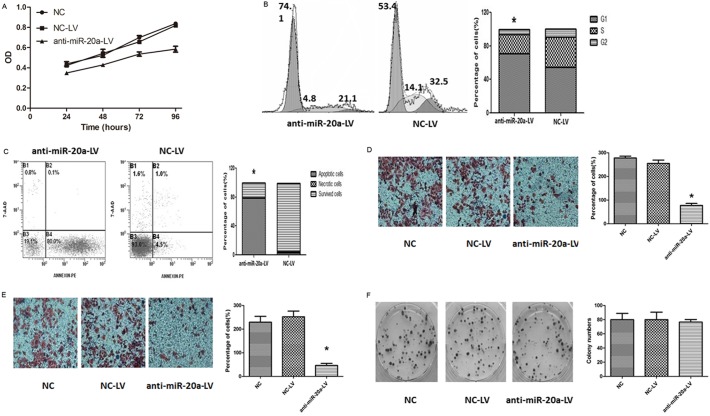
Cell growth characteristics, cell cycle, clone formation, cell migration, adhesion, cell invasion experiments were performed. A: Down-regulated miR-20a significantly inhibited cell proliferation in SiHa cells by MTT assay. B: Cell cycle determined by PI staining flow cytometry. C: Cell apoptosis detected by Annexin V-PE combined labeling flow cytometry, Apoptotic rate was increased and reached differences statistical significance in each group. D, E represented the results of cell invasion and migration across membrane with or without Matrigel, which showed that anti-miR-20a reduced the cell migration and invasion ability. F: The colony formation rates of these three groups had no significant difference.

Next, cell growth characteristics, cell cycle, clone formation, cell migration, adhesion, cell invasion experiments were performed. Growth curves showed that the infection of anti-miR-20a cervical cancer cell line growth slowed (Fig. 3A). Flow cytometry showed the average of G1 period of cells in the anti-miR-20a-LV group is 69.6%, while the corresponding control group was 55%, it is in reducing the proliferation cycle of cells (Fig. 3B). Apoptotic rate was increased and reached differences statistical significance in each group(*P*<0.001) (Fig. 3C). Migration, adhesion ability of invasion in cervical cancer cell was also detected, miR-20a could facilitate metastasis(*P*<0.01) (Fig. 3D and 3E). The colony formation rate of the two groups, was no significant difference (*P*>0.05) (Fig. 3F). As expected, down-expression of miR-20a significantly suppressed cell proliferation, migration and invasion abilities.

### MiR-20a Increases Tumor Growth In Vivo

**Fig 4:**
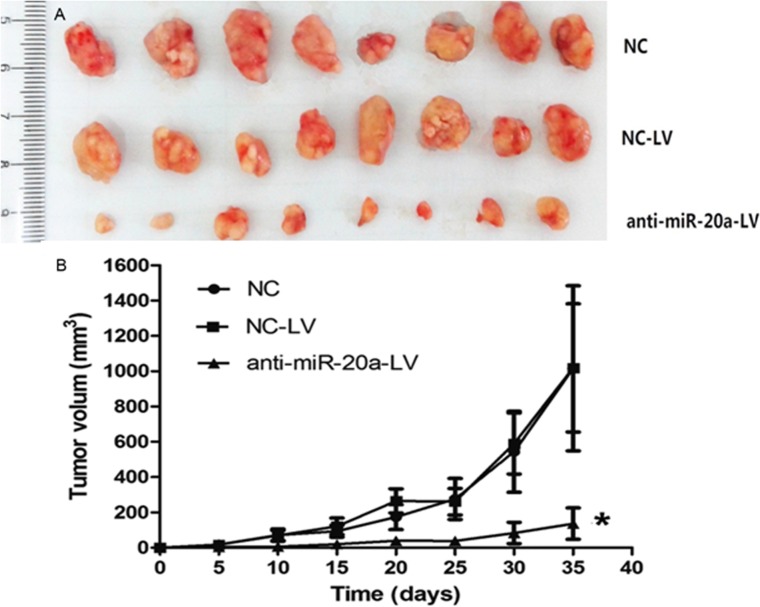
Inhibition of the growth of SiHa xenografts in nude mice by anti-miR-20a-LV. A: Human cervical cancer model transplanted subcutaneously in nude mice was established. At the end of the experiment, tumor volumes were measured to draw the growth curve of different groups. In subcutaneous tumor model the dimension of the tumor in experimental group is smaller than that in the control group. There was a significant difference among each group (*P*<0.01). B: Pictures of tumors in each group.

Based on the observed decreases in migratory, invasive and proliferative behaviors in SiHa cells infected with anti-miR-20a-LV, we next investigated the role of miR-20a in vivo. We subcutaneously inoculated nude mice with equal numbers (1×10^6^ cells per mouse) of SiHa cells with the forced expression of miR-20a or NC, Tumor incidence was assessed every five days, and tumors appeared in all the mice. The results showed that lentivirus mediated anti-miR-20a can significantly suppress the growth of cervical cancer xenografts in nude mice (Fig. 4).

### MiR-20a Directly Targets and Inhibits ATG7 and TIMP2

**Fig 5:**
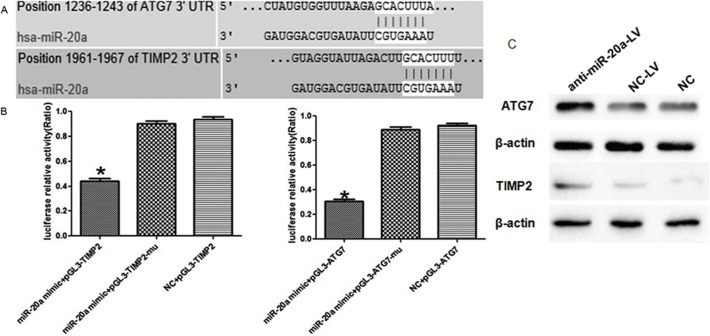
Targets validation of miR-20a in cervical cancer. A: Predicted consequential pairing of target region and miRNA by Targetscan, Pictar and Miranda. B: Luciferase activity displayed a significant decrease following miR-20a enforced expression compared with negative control group. C: Western blot analyses showed knockdown of miR-20a, through infection of anti-miR-20a-LV, in SiHa cells increased ATG7 and TIMP2 protein levels.

To understand how miR-20a facilitates cervical cancer growth and metastasis, we used three algorithms (Targetscan, Pictar and Miranda) to help identify miR-20a targets in human cervical cancers. Of these target genes that were predicted by all three algorithms (Fig. 5A), autophagy related protein 7(ATG7) and tissue inhibitors of metalloproteinase 2 (TIMP2) attracted our attention immediately as they have been implicated in tumorigenesis and metastasis. We cloned the ATG7 and TIMP2 3′-UTR into a luciferase reporter vector. Luciferase assay revealed that miR-20a directly bound to ATG7 and TIMP2 3′-UTR, and by which it remarkably reduced luciferase activities (Fig. 5B). However, mutation of the putative miR-20a sites in the 3′-UTR of ATG7 and TIMP2 abrogated luciferase responsiveness to miR-20a. To directly assess the effect of miR-20a on ATG7 and TIMP2 expression, we performed western blot analysis. As seen in Fig. 5C, knockdown of miR-20a, through infection of anti-miR-20a-LV, in SiHa cells increased ATG7 and TIMP2 protein levels. Taken together, these results indicate ATG7 and TIMP2 are direct downstream targets for miR-20a in SiHa cells. The above results prompted us to examine whether miR-20a suppresses cervical cancer growth and metastasis through repressing ATG7 and TIMP2 expression.

## Discussion

Our group has been focusing on the molecular mechanism of cervical squamous cell carcinoma development in recent years, especially devoting to the investigation of growth and metastasis. Lymph node metastasis(LNM) is the most major prognostic factor of the patients with Stage IB or IIA, the five-year survival rate of patients declines dramatically from approximately 80–95% in patients without lymph node metastases to approximately 50–65% in patients with positive lymph nodes. Therefore, we expected that the deregulation of microRNAs may facilitate the advanced progress of cervical squamous cell carcinoma. Zhang J, et al shed light on the negative feedback regulation of NF-κB/miR-130a/TNF-α/NF-κB in cervical cancer and may provide insight into the carcinogenesis of cervical cancer[5]. Wen SY, et al found that miR-506 expression was down-regulated in approximately 80% of the cervical cancer samples examined and inversely correlated with the expression of Ki-67[6]. Among oncogenic miRNAs, one of the best-characterized is miR-17-92, a polycistronic miRNA cluster, designated as OncomiR-1[7]. The precursor transcript contains six tandem stem-loop hairpin structures that ultimately yield six mature miRNAs: miR-17, miR-18a, miR-19a, miR-20a, miR-19b-1 and miR-92-1[8]. In particular, miR-17-92 is located at 13q31.3, a region amplified in several hematopoietic malignancies and solid tumors, including diffuse B-cell lymphomas, follicular lymphomas, Burkitt lymphomas, and lung carcinoma[9].

Recent findings indicate that these miRNAs are integrated components of the molecular pathways that regulate tumor development and tumor maintenance, the effects of miR-20a overexpression have been examined in multiple animal models, human cancers, and cell culture systems for its ability to regulate a number of cellular processes that favor malignant transformation[10–11]. These studies as a whole revealed that miR-20a functions pleiotropically during both normal development and malignant transformation to promote proliferation, inhibit differentiation, augment angiogenesis, and sustain cell survival. However, the potential function of this microRNA in human cervical squamous cell carcinoma progression has few reports. In the present study, we are interested in the potential role of miR-20a in the malignant progression of SiHa cells.

In this study, we found that miR-20a was frequently up-regulated in tissue specimens. Thus, we supposed that miR-20a may be a novel tumor oncogene miRNA and its deregulation may involve the advanced progress of human cancer. Next, we investigated the function of miR-20a in SiHa cells. Establish the stable anti-miR-20a cervical cancer cell lines by lentivirus, and precede to cell growth characteristics, cell cycle, clone formation, cell migration, adhesion, and invasion experiments. The model of down-expressed miR-20a cervical cancer xenografts in nude mice was also established which explore the potential of anti-miR-20a in vivo. Then we found that inhibited miR-20a prevented tumor progression by modulating cell cycle, proliferation, apoptosis, and invasion. Growth curves showed that the infection of anti-miR-20a cervical cancer cell line growth slowed. The model of down-expressed miR-20a cervical cancer xenografts in nude mice was established, the results showed that lentivirus mediated anti-miR-20a can significantly suppress the growth of cervical cancer xenografts in nude mice.

We further characterized ATG7 and TIMP2 as functional targets of miR-20a by luciferase reporter gene assays and western blot analysis, respectively. Autophagy delivers cytoplasmic components to lysosomes for degradation and recycling of the degradation products, such as amino acids, carbohydrates and lipids that are used to synthesize new proteins and organelles or metabolized to supply energy[12]. Autophagy is intimately associated with eukaryotic cell death and apoptosis. Indeed in some cases the same proteins control both autophagy and apoptosis. Apoptotic signaling can regulate autophagy and conversely autophagy can regulate apoptosis. It has been proposed that “autophagic cell death” is another type of “active” programmed death[13]. ATG7 is one of the master regulators of the autophagy process, responsible for two major reactions involved in autophagosome formation and in vesicle progression[14]. Moreover, it was already established that Atg7−/− knockout mice die within one day from birth and show reduced pup size, due to an impaired autophagy pathway[8]. Yoshihiro Inami, et al found that loss of Atg7 in mouse liver causes hepatocellular adenoma[15]. Through the use of gain-or-lose molecules, we demonstrated that miR-20a was able to modulate autophagy varying endogenous ATG7 expression levels, and this effect was mediated via a miR-20a consensus sequence contained in the 3′-UTR of the gene.

During the process of tumor invasion, essential steps include the degradation of basement membranes and remodeling of the extracellular matrix (ECM) by proteolytic enzymes such as matrix metalloproteinases (MMPs) under regulation by tissue inhibitors of metalloproteinases (TIMPs). MMPs, particularly MMP-2 and MMP-9, are key enzymes known to degrade the components of surrounding ECM during cancer invasion and metastasis[16]. The computational prediction of miRNAtarget sites suggested TIMP2 maybe the target of miR-20a, which was verified by westernblot and luciferase assay also.

## Conclusions

Our results suggest that miR-20a expression may be related with malignant process of cervical cancer, especially invasion and metastasis by targeting ATG7 and TIMP2. Large-scale and long-term follow-up studies are needed to confirm the significance of miR-20a in cervical cancer. MiRNAs may be an attractive target for therapeutic intervention.

## Supporting Information

S1 Fig Vector map. A: Lentivirus Vector map; B pGL3 promoter vector map.
(DOC)

S2 Fig Lentivirus Vector sequencing results. (DOC)

S1 Dataset Target gene sequences by chemical synthesis. Both sides of the coding sequences were XbaI Restriction Enzyme cutting sites, labelled zones were binging sites.
(DOC)

S1 Table Association between the expression of miR-20a with clinicopathological features in patients with cervical cancer. (DOC)

S2 Table OD value after infection(MTT). (DOC)

S3 Table Inhibition of the growth of SiHa xenografts in nude mice by anti-miR-20a-LV. (DOC)
