# Supplementary figures and images for: MiR-20a Promotes Cervical Cancer Proliferation and Metastasis In Vitro and In Vivo
Source: PLoS One. 2015 Mar 24;10(3):e0120905. doi: 10.1371/journal.pone.0120905 (PMC4372287; doi:10.1371/journal.pone.0120905)

**A**


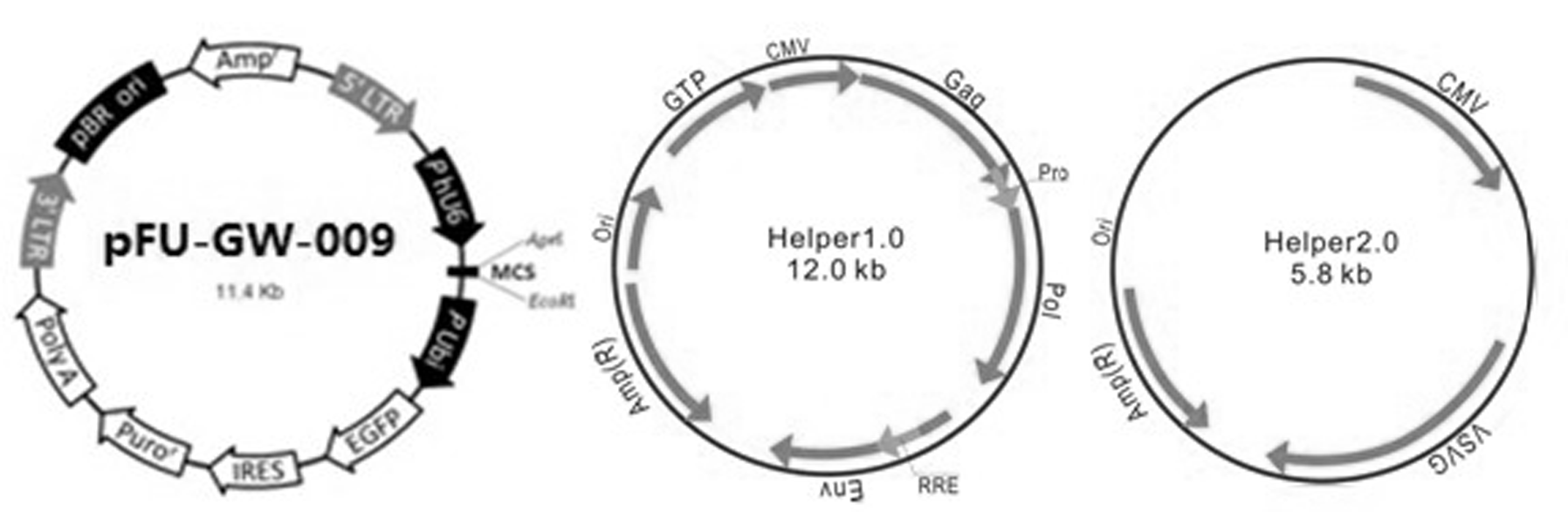


**B**


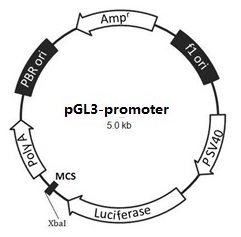


**S1_Fig : Vector map .** A: Lentivirus Vector map; B pGL3 promoter vector map.

Supplement: S1 Fig — A: Lentivirus Vector map; B pGL3 promoter vector map. (DOC) [file pone.0120905.s001.doc]
