# Supplementary material for: MiR-20a Promotes Cervical Cancer Proliferation and Metastasis In Vitro and In Vivo
Source: PLoS One. 2015 Mar 24;10(3):e0120905. doi: 10.1371/journal.pone.0120905 (PMC4372287; doi:10.1371/journal.pone.0120905)
Supplement: S2 Fig — (DOC) [file pone.0120905.s002.doc]

GAGATATTGGAATTAATTTGACTGTAAACACAAAGATATTAGTACAAAATACGTGACGTAGAAAGTAATAATTTCTTGGGTAGTTTGCAGTTTTAAAATTATGTTTTAAAATGGACTATCATATGCTTACCGTAACTTGAAAGTATTTCGATTTCTTGGCTTTATATATCTTGTGGAAAGGACGAAACACCGGCTACCTGCACTATAAGCACTTTATTTTTGAATTCTCGACCTCGAGACAAATGGCAGTATTCATCCACGGATCCTAACCCGTGTCGGCTCCAGATCTGGCCTCCGCGCCGGGTTTTGGCGCCTCCCGCGGGCGCCCCCCTCCTCACGGCGAGCGCTGCCACGTCAGACGAAGGGCGCAGCGAGCGTCCTGATCCTTCCGCCCGGACGCTCAGGACAGCGGCCCGCTGCTCATAAGACTCGGCCTTAGAACCCCAGTATCAGCAGAAGGACATTTTAGGACGGGACTTGGGTGACTCTAGGGCACTGGTTTTCTTTCCAGAGAGCGGAACAGGCGAGGAAAAGTAGTCCCTTCTCGGCGATTCTGCGGAGGGATCTCCGTGGGGCGGTGAACGCCGATGATTATATAAGGACGCGCCGGGTGTGGCACAGCTAGTTCCGTCGCAGCCGGGATTTGGGTCGCGGTTCTTGTTTGTGGATCGCTGTGATCGTCACTTGGTGAGTAGCGGGCTGCTGGGCTGGCCGGGGCTTTCGTGGCCGCCGGGCCGCTCGGTGGGACGGAAGCGTGTGGAGAGACCGCCAAGGGCTGTAGTCTGGGTCCGCGAGCAAGGTTGCCCTGAACTGGGGGTTGGGGGGAGCGCAGCAAAATGGCGGCTGTTCCCGAGTCTTGAATGGAAGACGCTTGTGAGGCGGGCTGTGAGGTCGTTGAAACAAGGTGGGGGGCATGGTGGGCGGCAAGAACCCAAGGTCTTGAGGCTTCGCTAATGCGGGAAAGCTCTTATTCGGTGAGATGGGCTGGGGCACCATCTGGGGACCTTGACGTGAAGG


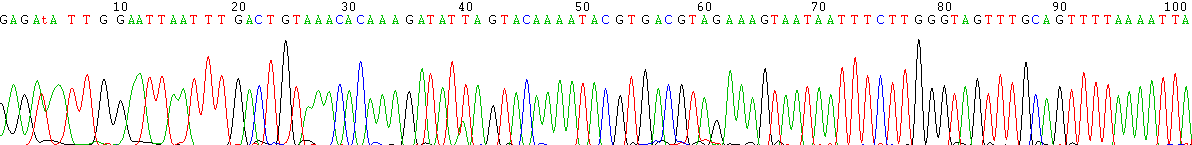


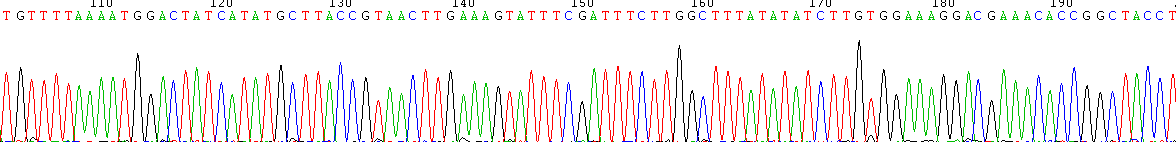


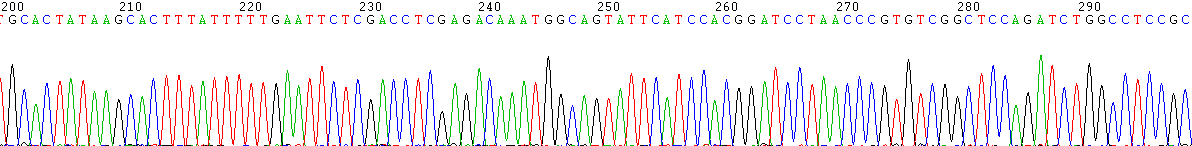


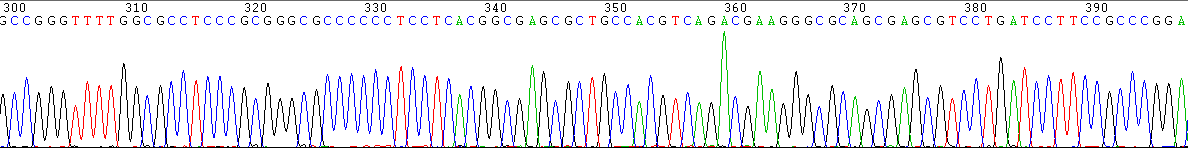

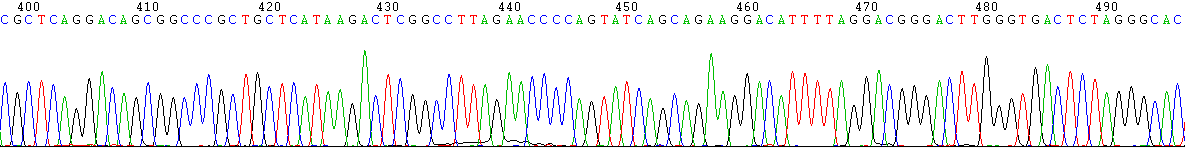


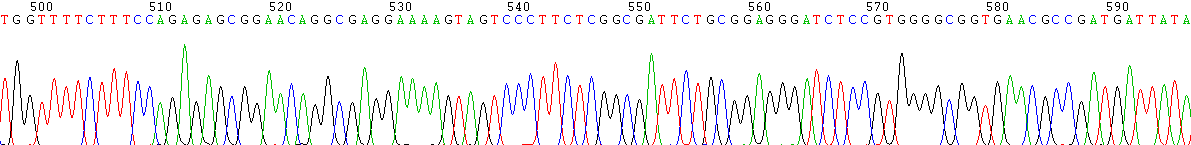


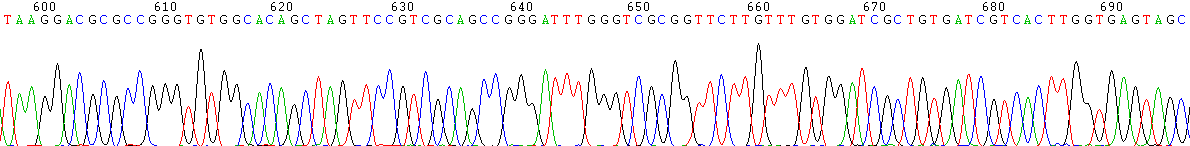


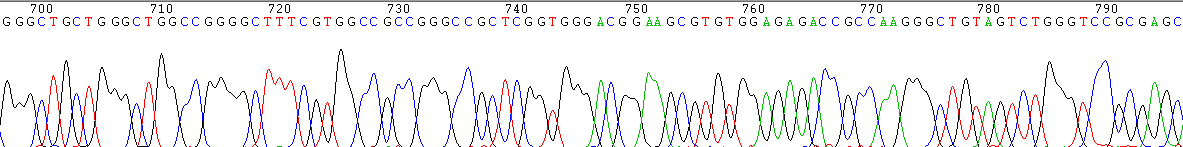


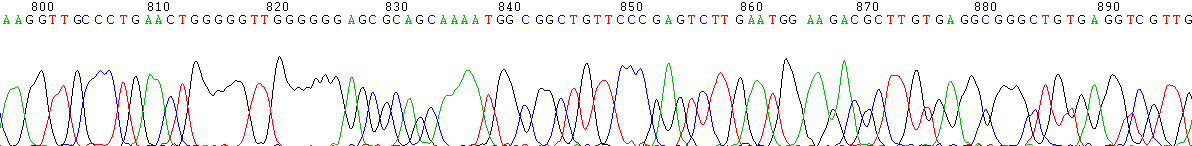


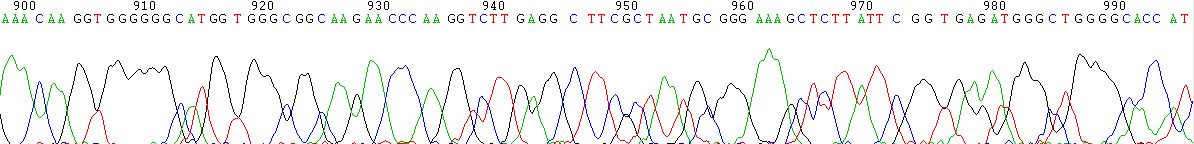


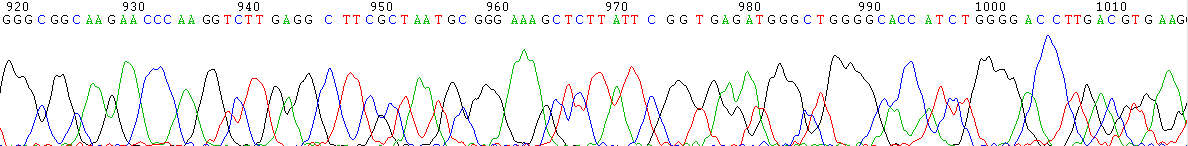


**S2_Fig: Lentivirus Vector sequencing results**
