# Supplementary material for: MiR-20a Promotes Cervical Cancer Proliferation and Metastasis In Vitro and In Vivo
Source: PLoS One. 2015 Mar 24;10(3):e0120905. doi: 10.1371/journal.pone.0120905 (PMC4372287; doi:10.1371/journal.pone.0120905)
Supplement: S1 Dataset — Both sides of the coding sequences were XbaI Restriction Enzyme cutting sites, labelled zones were binging sites. (DOC) [file pone.0120905.s003.doc]

① TIMP2（NM_003255-3UTR）

TCTAGATTCTGCTGATTGTTTTTTTAATGTTTTGGTTTGTTTTTGACATCAGCTGTAATCATTCCTGTGCTGTGTTTTTTATTACCCTTGGTAGGTATTAGACTTgcactttTTTAAAAAAAGGTTTCTGCATCGTGGAAGCATTTGACCCAGAGTGGAACGCGTGGCCTATGCAGGTGGATTCCTTCAGGTCTTTCCTTTGGTTCTTTGAGCTCTAGA

② TIMP2（NM_003255-3UTR-mu）

TCTAGATTCTGCTGATTGTTTTTTTAATGTTTTGGTTTGTTTTTGACATCAGCTGTAATCATTCCTGTGCTGTGTTTTTTATTACCCTTGGTAGGTATTAGACTTtatagggTTTAAAAAAAGGTTTCTGCATCGTGGAAGCATTTGACCCAGAGTGGAACGCGTGGCCTATGCAGGTGGATTCCTTCAGGTCTTTCCTTTGGTTCTTTGAGCTCTAGA

③ ATG7（NM_ 001136031-3UTR）

TCTAGACATGGGAGCTTCATGGGGACACAGCCGGCACAGGTGCAGGGCCCGAGTCCGCCCACCCAGCCTGGCGCTGAAACTGCACACGTACACTATGTGGTTTAAGAgcactttaTTATTGTTCTTAAGGCTACTTTTAAGTACAAAAAAAGATGGCCTGCCAAACCTTTTTTTTTCTTCTTCCAGGAAAAACAGGCCACAGAGAATGGTATATTACAGATTTTCTAGA

④ ATG7（NM_ 001136031-3UTR-mu）

TCTAGACATGGGAGCTTCATGGGGACACAGCCGGCACAGGTGCAGGGCCCGAGTCCGCCCACCCAGCCTGGCGCTGAAACTGCACACGTACACTATGTGGTTTAAGAtatagggtTTATTGTTCTTAAGGCTACTTTTAAGTACAAAAAAAGATGGCCTGCCAAACCTTTTTTTTTCTTCTTCCAGGAAAAACAGGCCACAGAGAATGGTATATTACAGATTTTCTAGA

**S1_Dataset: Target gene sequences by chemical synthesis.** Both sides of the coding sequences were *Xba*I Restriction Enzyme cutting sites, labelled zones were binging sites.
