# Supplementary material for: MiR-20a Promotes Cervical Cancer Proliferation and Metastasis In Vitro and In Vivo
Source: PLoS One. 2015 Mar 24;10(3):e0120905. doi: 10.1371/journal.pone.0120905 (PMC4372287; doi:10.1371/journal.pone.0120905)
Supplement: S1 Table — (DOC) [file pone.0120905.s004.doc]

**S1_Tab Association between the expression of miR-20a with clinicopathological features in patients with cervical cancer**

|  | **N** | **Fold change of**  **miR-20a**  **(median^*^)** |
| --- | --- | --- |
| **Age**  ＜50  ≥50  ***P***  **Diameter of tumor**  ＜4cm  ≥4cm  ***P***  **FIGO stage**  Ⅰ  Ⅱ  Ⅲ  Ⅳ  ***P***  **Histologic grade**  Well  Moderate  Poorly  ***P***  **LNM**  stageⅠ  negative  positive  ***P***  stageⅡA  negative  positive  ***P*** | 60  40  83  17  51  35  9  5  61  18  21  28  23  12  17 | 6.94(1.71-55.8)  6.92(1.7-27.786)  **0.602**  3.56(1.7-27.8)  55.8(6.9-224.9)  **0.013**  1.74(0.9-27.3)  6.97(1.73-28.28)  27.83(17.1-83.6)  226(113-666)  **0.004**  3.51(1.3-27.6)  10.39(0.4-34.6)  28.01(4.3-110.6)  **0.027**  1.72(0.54-6.05)  13.75(0.9-111.5)  **0.001**  1.74(0.55-6.08)  27.5(1.3-55.8)  **0.001** |
