# Supplementary material for: MiR-20a Promotes Cervical Cancer Proliferation and Metastasis In Vitro and In Vivo
Source: PLoS One. 2015 Mar 24;10(3):e0120905. doi: 10.1371/journal.pone.0120905 (PMC4372287; doi:10.1371/journal.pone.0120905)
Supplement: S2 Table — (DOC) [file pone.0120905.s005.doc]

**S2_Tab OD value after infection(MTT)**

|  | OD value | | | |
| --- | --- | --- | --- | --- |
| Group | 24h | 48h | 72h | 96h |
| anti-miR-20a-LV | 0.348±0.03 | 0.429±0.03 | 0.536±0.1 | 0.584±0.12 |
| NC-LV | 0.423±0.03 | 0.543±0.02 | 0.657±0.06 | 0.819±0.08 |
| NC | 0.439±0.05 | 0.523±0.03 | 0.699±0.04 | 0.838±0.07 |
