# Supplementary material for: MiR-20a Promotes Cervical Cancer Proliferation and Metastasis In Vitro and In Vivo
Source: PLoS One. 2015 Mar 24;10(3):e0120905. doi: 10.1371/journal.pone.0120905 (PMC4372287; doi:10.1371/journal.pone.0120905)
Supplement: S3 Table — (DOC) [file pone.0120905.s006.doc]

**S3_Tab Inhibition of the growth of SiHa** **xenografts in nude mice by anti-miR-20a-LV**

| Group | Volumes（mm^3^） | Inhibition rate |
| --- | --- | --- |
| NC  NC-LV  anti-miR-20a-LV | 1016±165.3  1019±128.4  136.5±31.43 | 0  0  86.6% |
